# Neutrophil-to-Lymphocyte Ratio as a Marker for Cardiac Autonomic Neuropathy in Egyptian Patients With Type 2 Diabetes: A Cross-Sectional Study

**DOI:** 10.7759/cureus.61634

**Published:** 2024-06-04

**Authors:** Samir H Assaad-Khalil, Talaat Abdel Aaty, Mohamed El Feky, Hoda Mohamed Abdel Naby, Nada Ramadan El Essawy, Noha Gaber Amin

**Affiliations:** 1 Internal Medicine, Unit of Diabetes, Lipidology & Metabolism, Faculty of Medicine, Alexandria University, Alexandria, EGY; 2 Cardiology, Faculty of Medicine, Alexandria University, Alexandria, EGY; 3 Rehabilitation & Physical Medicine, Faculty of Medicine, Alexandria University, Alexandria, EGY

**Keywords:** neutrophil-to-lymphocyte ratio, platelet-to-lymphocyte ratio, diabetes mellitus type 2, diabetic peripheral neuropathy, cardiac autonomic neuropathy

## Abstract

Objective: Cardiac autonomic neuropathy (CAN) is one of the most serious complications of diabetes. This study aimed to analyze the correlation between neutrophil-to-lymphocyte ratio (NLR) and CAN in patients with type 2 diabetes (T2D) using 24-hour Holter ECG and to assess the relationship between NLR and severity of diabetic peripheral neuropathy (DPN).

Subjects & methods: This cross-sectional study included 90 T2D patients with DPN confirmed by nerve conduction study (NCS). A 24-hour Holter ECG was done to detect the decrease in heart rate variability (HRV). Laboratory parameters, including fasting blood glucose, creatinine, cholesterol, triglyceride, and glycosylated hemoglobin (HbA1c) levels, as well as CBC, neutrophils, lymphocytes, NLR, and platelet-to-lymphocyte ratio (PLR), were calculated accordingly. An albumin‐to‐creatinine ratio (ACR) test was done and the estimated glomerular filtration rate (eGFR) was calculated. Chronic kidney disease was diagnosed by the presence of albuminuria (≥30 mg/g creatinine) and/or eGFR less than 60.

Results: Based on the 24-hour Holter ECG, 25 patients out of 90 (27.7%) had CAN. On comparing both the CAN and non-CAN groups, the CAN group had higher HbA1C (p = 0.005), higher NLR (p = 0.014), and higher neutrophils (p = 0.10). Also, PLR was higher in the CAN group than in the non-CAN group, but this was not statistically significant (p = 0.180). Receiver operator characteristic curve analysis revealed that NLR with a cutoff of 1.7 succeeded in detecting patients with CAN.

Conclusion: NLR can be used as an inexpensive and accessible marker to detect patients with diabetes at risk for developing CAN.

## Introduction

In 2021, 537 million adults (20-79 years) were living with diabetes, and by 2045, this will increase to 783 million [1]. The prevalence of type 2 diabetes (T2D) globally is expected to increase to 7,079 per 100,000 people by 2030 [2].

Chronic hyperglycemic state in T2D triggers the initiation and development of non-enzymatic glycation reactions with lipids, proteins, and nucleic acids. Therefore, microvascular and macrovascular complications arise from increased accumulation of advanced glycation end products in the glomerulus, retina, and major blood vessel wall. Chronic hyperglycemia leads to oxidative stress in the vessel walls through the oxidation of low-density lipoprotein, which increases the release of cytokines and growth factors and changes in cell adhesion, as the inflammatory response plays a major role in diabetic complications. These factors are very costly to be used as a screening tool. Thus, there is a need to find a reliable marker for the early detection of diabetic complications [3].

Cardiac autonomic neuropathy (CAN) is one of the most serious complications of diabetes, which is not easily recognized by the patients. It increases the morbidity and mortality significantly in patients with diabetes. The prevalence of CAN in type 1 diabetes (T1D) patients ranges from 1% to 90% while the prevalence of CAN in T2D patients ranges from 20% to 73% [4].

Clinical manifestations of CAN vary from resting tachycardia, loss of circadian rhythm of blood pressure, orthostatic hypotension, and exercise intolerance to malignant cardiac arrhythmia, silent myocardial infarction, and sudden death. Usually, in the late stages of diabetes, the symptoms of CAN appear, but a subclinical CAN could be detected in patients with diabetes one year after the initial diagnosis [5].

The vagus nerve, which is responsible for about 75% of parasympathetic activity, is the first to be affected by diabetes and this leads to parasympathetic denervation and sympathetic tone predominance. Later on, sympathetic nerves are affected leading to sympathetic function damage [6]. A decrease in heart rate variability (HRV) is the earliest sign of CAN and could be detected at the subclinical stage. HRV is a result of continuous alterations in the heart's parasympathetic and sympathetic output, and this could be assessed by ECG.

Diabetic peripheral neuropathy (DPN) is considered one of the most common complications of diabetes and may lead to diabetic foot lesions like infection, ulcers, gangrene, and amputation. These complications are related to increased mortality and worse quality of life, as well as a large cost to healthcare systems. As a result, finding a reliable biomarker for the early detection of DPN is critical [7].

The neutrophil-to-lymphocyte ratio (NLR) is a cheap, widely accessible, and reliable biomarker that could be used for subclinical inflammation diagnosis with the elevation of other pro-inflammatory cytokines. Both systemic lymphopenia and neutrophilia are associated with a worse prognosis in a variety of inflammatory, viral, and cardiovascular disorders, as well as malignancies [8].

An increased neutrophil count indicates a continuous, nonspecific, damaging inflammatory activity, whereas a low lymphocyte count suggests comparatively insufficient immunological control and dormant immune pathways [9]. As a result, increased NLR can indicate the immune system’s functioning condition during chronic inflammation. Numerous studies have established its significance in systemic inflammation in diabetes.

NLR is also believed to be indicative of an autonomic vascular imbalance. The sympathetic nerves stimulate granulocyte release, whereas the parasympathetic nerves stimulate lymphocyte release [10].

The objective of this study was to assess the relationship between NLR and CAN in T2D patients, to detect the cut-off point of NLR for the development of CAN, and to assess the correlation between NLR and the severity of DPN.

## Materials and methods

Subjects & methods

This is a cross-sectional study that included 90 patients whose ages ranged from 21 to 65 years, had T2D (diagnosed according to the American Diabetes Association's (ADA) and the World Health Organization's diagnostic criteria for T2D diagnosis), and had DPN confirmed with nerve conduction study (NCS) in addition to any symptom or sign of DPN as recommended by the Toronto Neuropathy Expert Group for the diagnosis of DPN [11]. Patients were recruited from the Diabetes, Lipidology & Metabolism Unit’s outpatient clinic at Alexandria Main University Hospitals in Egypt in the duration between November 2022 and March 2023.

A sample size of 90 subjects was calculated, taking into consideration a 95% confidence level and 80% power using the chi-square test to detect an odds ratio (OR) ≥ 3.5.

We excluded patients with T1D, thyroid disease, other causes of neuropathy (such as vitamin B12 deficiency, drug-induced neuropathy, and renal failure), diabetic foot ulcers, acute illness, malignancy, cerebrovascular stroke, coronary heart disease, patients who receive drugs that affect heart rate as beta-blockers, severe neurological disease, and hematological diseases.

Ethical approval

The study was approved by the Institutional Ethics Committee of Alexandria University (IRB approval number: 00012098; dated: 6-10-2022), which is done in accordance with the Declaration of Helsinki 1964 and its later amendments. Before any study-related procedure, each subject signed an informed consent at the beginning of the study.

Data collection

History-Taking and Full Clinical Examination

Complete medical history was taken from all 90 subjects, including their age, sex, duration of diabetes, medications, symptoms of DPN as numbness or prickling sensation, and symptoms of CAN as lightheadedness or blurring of vision. Anthropometric measures were assessed, including weight, height, waist circumference, and BMI. Clinical examination included resting heart rate and blood pressure recording in supine and standing positions to detect postural hypotension, which is defined as a drop >20 mmHg in systolic pressure or >10 mmHg in diastolic blood pressure in response to a change in posture from supine to standing.

Laboratory Analyses

Venous blood samples (5 mL from each patient) were collected. Sampling was done in the morning after an overnight fast, and routine chemical and hematological tests were performed using automated analyzers. Laboratory investigations included CBC with differential, including neutrophils and lymphocytes. NLR is calculated by dividing the absolute neutrophil ratio by the absolute lymphocyte ratio, and the platelet-to-lymphocyte ratio (PLR) is calculated by dividing the number of platelets by the absolute lymphocyte ratio. Also, we measured fasting blood glucose, glycosylated hemoglobin (HbA1c), serum creatinine, estimated glomerular filtration rate (eGFR), lipid profile, serum glutamic pyruvic transaminase (SGPT), serum glutamic oxaloacetic transaminase (SGOT), and albumin‐to‐creatinine ratio (ACR) in urine. A patient was defined to have chronic kidney disease (CKD) if there is albuminuria (≥30 mg/g creatinine) and/or eGFR less than 60.

Assessment of DPN

Vibration perception threshold (VPT) was measured using a neurothesiometer (Vibration Perception Threshold Meter device; INPUT:100-130 V ~ 60 HZ 30W; Diabetica Solutions Inc, San Antonio, TX). VPT in both feet of value 16V or more is considered as DPN [12]. We used 10-g Semmes-Weinstein monofilament, which puts 10 g force using a 5.07 Semmes-Weinstein nylon monofilament on 10 locations on each lower limb avoiding areas with scars, ulcers, necrotic, and calluses. It was considered abnormal if at least two locations in one foot could not be felt [13].

Neuropathy was also assessed using different scores, including the neuropathy symptom score (NSS), which is a validated symptom score consisting of four items with a high predictive value for DPN screening related to diabetes. The presence of neuropathic pain, paresthesia, numbness, or instability when walking counts as one point toward the maximum score of four. DPN is defined as having a score of one or more [14]. Neuropathy disability score (NDS) is a sum of parameters, and a score >3 was considered diagnostic of DPN. Three parameters (temperature perception, VPT, and pinprick sensation) were rated as normal (0) or abnormal (1), while Achilles reflexes were scored as present (0), present with reinforcement (1), and absent (2) [14].

Nerve Conduction Studies (NCS)

Peripheral neuropathy was diagnosed based on standard NCS. Nerve conduction velocity (NCV), latency, and amplitude were measured using a Neuropack 2 electromyography apparatus (MEB-7102K and MEB-9400) from Nihon Kohden (Tokyo, Japan). The temperature in the room in which the electrophysiological studies were done was not less than 22°C. Surface recording electrodes of 0.6 mm diameter were used to take measurements in the upper and lower limbs. Electrical pulses lasting one millisecond and repeated every second were used to stimulate nerves. The intensity of the pulses was strong enough to generate the highest amplitude in both the action potential of a compound muscle and a sensory nerve [15]. Velocity, latency, and amplitude were recorded in both the upper and lower limbs’ sensory and motor nerves, left ulnar, sural nerves, right median, right peroneal, and left tibial nerves.

Assessment of CAN

According to the Toronto Consensus Panel, the North American Society of Pacing and Electrophysiology, the European Society of Cardiology, and the ADA position statement on diabetic neuropathy recommend the following about CAN assessments for clinical trials, measuring a targeted intervention, or for prognostication (Task Force of the European Society of Cardiology and the North American Society of Pacing and Electrophysiology): using indices of HRV, including the root mean square of the difference of successive R-R intervals (rMSSD) and standard deviation of all regular R-R intervals (SDNN), and using 24 hour Holter monitoring [16].

R-R recordings lasting 24 hours allow the computation of more intricate statistical time domain metrics, such as SDNN, rMSSD, SD of a five-minute average of regular R-R intervals (SDANN), and the number of times per hour in which two successive R-R intervals differ by >50 ms over 24 hours (pNN50). SDNN represents the combined parasympathetic and sympathetic modulation of HRV, while pNN50 and rMSSD represent the parasympathetic limb. Normative values are defined as previously reported [16].

DPN was diagnosed based on NCS, while CAN was diagnosed based on 24-hour Holter ECG (SDNN and RMSSD).

Examination of peripheral pulsations was done and ankle-brachial index (ABI) was calculated using a standardized Doppler ultrasonic device (5 MHz; Nicolet Elite 200 R, VIASYS Healthcare Inc., Madison, WI). Peripheral arterial disease was considered if ABI was ≤0.9 in any lower limb.

Statistical analysis

Data were analyzed using IBM SPSS software package version 20.0 (IBM Corp., Armonk, NY) [17]. Qualitative data were illustrated using numbers and percentages. Quantitative data were presented as mean and standard deviation for normally distributed variables and for non-normally distributed variables, data were shown as median and interquartile range (IQR). The Kolmogorov-Smirnov and Shapiro-Wilk tests were utilized to verify the normality of distribution. Student's t-test was used for quantitative variables with a normal distribution to distinguish between the two groups under study. Mann-Whitney test was used for quantitative variables with abnormal distribution to distinguish between the two groups under study. Receiver operating characteristics (ROC) curve analysis was done to determine the NLR cutoff point, which has the highest sensitivity and specificity, and the significance of the obtained results was judged at the 5% level.

## Results

Our study included 90 patients with T2D and DPN, of whom 25 patients (27.8%) had CAN, diagnosed by decreased HRV by 24-hour Holter ECG.

On comparing both CAN and non-CAN groups, there was no statistically significant difference between them regarding age, duration of diabetes, waist circumference, and BMI (Table 1).

**Table 1:** Comparison between CAN and non-CAN groups regarding different variables (n = 90). p: p-value for comparing the three studied groups; *: statistically significant at p ≤ 0.05; t: Student's test; U: Mann-Whitney. CAN: cardiac autonomic neuropathy; BMI: body mass index; VPT: vibration perception threshold; NDS: neuropathy disability score; NSS: neuropathy symptom score; ABI: ankle-brachial index.

Parameters	24-hour Holter ECG				Test of sig.	P
	Non-CAN (n = 65)		CAN (n = 25)			
Sex						
Male	21 (32.3%)		5 (20%)		1.331	0.249
Female	44 (67.7%)		20 (80%)			
Age (years)						
Mean ± SD	54.66 ± 6.61		53.40 ± 7.30		t = 0.787	0.433
Duration (years)						
Mean ± SD	12.51 ± 7.41		13.68 ± 5.96		U=697.00	0.294
Symptoms of CAN						
No	21 (32.3%)		7 (28%)		0.156	0.693
Yes	44 (67.7%)		18 (72%)			
Postural hypotension						
Yes	39 (60%)		21 (84%)		4.68	0.031*
No	26 (40%)		4 (16%)			
Supine blood pressure (mmHg)						
Systolic						
Mean ± SD	135.4 ± 17.19		138.8 ± 17.34		0.842	0.402
Diastolic					t = 0.110	0.913
Mean ± SD	90.31 ± 11.72		90.0 ± 12.25			
Standing blood pressure (mmHg)						
Systolic					t = 0.102	0.919
Mean ± SD	121.5 ± 19.04		122.0 ± 19.79			
Diastolic					t = 1.198	0.234
Mean ± SD	81.0 ± 14.90		77.0 ± 12.08			
Pulse (beats/min)						
Mean ± SD	91.42 ± 12.67		97.76 ± 12.01		2.158*	0.034*
BMI (kg/m2)						
Mean ± SD	33.55 ± 6.24		33.68 ± 4.47		t = 0.096	0.924
Waist circumference (cm)						
Mean ± SD	117.7 ± 11.90		117.5 ± 8.83		t = 0.054	0.957
VPT						
Right						
Mean ± SD	38.65 ± 17.24		39.28 ± 18.09		U = 794.0	0.867
Left						
Mean ± SD	38.65 ± 17.24		39.28 ± 18.09		U = 794.0	0.867
ABI						
Right						
Mean ± SD	1.06 ± 0.09		1.07 ± 0.10		t = 0.581	0.562
Left						
Mean ± SD	1.06 ± 0.09		1.07 ± 0.10		t = 0.581	0.562
NDS						
Mean ± SD	9.54 ± 0.85		9.36 ± 1.25		t = 0.009	0.926
NSS						
Mean ± SD	3.42 ± 0.83		3.48 ± 0.87		U = 755.0	0.551

Regarding clinical examination, 84% of the CAN group had postural hypotension, while 60% of patients in the non-CAN group had postural hypotension (p = 0.031). Also, there was a significant difference between the two groups regarding resting heart rate (p = 0.034), with a mean of 97.76 ± 12.01 in the CAN group and 91.42 ± 12.67 in the non-CAN group.

Regarding the neurological clinical examination, VPT was slightly higher in the CAN group than in the non-CAN group, but this was not statistically significant. Also, there was no statistically significant correlation between both groups regarding the scores of NDS and NSS.

Comparison between the two groups regarding laboratory investigations

Regarding laboratory investigations, HbA1C was significantly higher in the CAN group (p = 0.005), and ACR was slightly higher in the CAN group with a mean of 74.19 ± 159.1 mcg/mg in comparison to the non-CAN group (39.24 ± 85.28 mcg/mg) (p = 0.030) (Table 2).

**Table 2:** Comparison of laboratory investigations between the CAN and non-CAN groups (n = 90). p: p-value for comparing between the three studied groups; *: statistically significant at p ≤ 0.05; t: Student's test; U: Mann-Whitney. CAN: cardiac autonomic neuropathy; HbA1C: glycated hemoglobin; eGFR: estimated glomerular filtration rate; UACR: urinary albumin‐to‐creatinine ratio; TC: total cholesterol; LDL: low-density lipoproteins; HDL: high-density lipoproteins; TG: triglycerides.

Parameters	24-hour Holter ECG		Test of sig.		p
	Non-CAN (n = 65)	CAN (n = 25)			
HbA1C (%)					
Mean ± SD	8.44 ± 1.71	9.71 ± 2.28	t = 2.871^*^		0.005^*^
Fasting blood glucose (mg/dl)					
Mean ± SD	156.5 ± 64.19	190.6 ± 87.62	U = 656.0		0.159
eGFR					
Mean ± SD	96.22 ± 31.32	115.8 ± 28.35	t = 2.723^*^		0.008^*^
UACR (mg/g)					
Mean ± SD	39.24 ± 85.28	74.19 ± 159.1	U = 572.0^*^		0.030^*^
TC (mg/g)					
Mean ± SD	182.9 ± 58.51	194.8 ± 47.72	U = 647.0		0.136
LDL (mg/g)					
Mean ± SD	103.5 ± 38.05	115.3 ± 41.30	U = 669.0		0.196
HDL (mg/g)					
Mean ± SD	45.06 ± 12.56	47.04 ± 9.60	U = 692.0		0.277
TG (mg/g)					
Mean ± SD	168.5 ± 196.3	160.3 ± 57.54	U = 686.0		0.254

Regarding nerve conduction parameters, DPN was objectively diagnosed in the studied patients; however, there was no statistically significant difference between the two groups regarding the sensory parameters, while in the motor parameters, there was a statistically significant correlation between both groups in NCV of the right peroneal nerve, showing a lower range of 0.0-61.90 m/s and a mean ± SD of 37.0 ± 13.70 m/s in the CAN group while in the non-CAN group, it showed a range of 0.0-72.30 and mean ± SD of 43.59 ± 11.21 m/s (p = 0.034). Also, distal latency (DL) of the left posterior tibial nerve in the CAN group was more delayed with a range of 3.96-40.3 ms and mean ± SD of 7.50 ± 6.97 ms while in the non-CAN group, it ranged from 3.28 to 10.40 ms, with a mean ± SD of 5.59 ± 1.50 ms (p = 0.042), as shown in Table 3.

**Table 3:** Comparison between the CAN and non-CAN groups regarding nerve conduction study (n = 90). p: p-value for comparing the two studied groups; *: statistically significant at p ≤ 0.05. CAN: cardiac autonomic neuropathy; NCS: nerve conduction study.

	24-hour Holter ECG		Test of sig.	p
	Non-CAN (n = 65)	CAN (n = 25)		
Peroneal motor NCS (right)				
Distal latency in ms				
Mean ± SD	5.71 ± 1.46	5.54 ± 2.62	739.00	0.508
Amplitude in millivolts				
Mean ± SD	3.93 ± 6.21	4.86 ± 10.86	691.50	0.275
Nerve conduction velocity in m/s				
Mean ± SD	43.59 ± 11.21	37.0 ± 13.70	577.50^*^	0.034^*^
Posterior tibial motor NCS (left)				
Distal latency in ms				
Mean ± SD	5.59 ± 1.50	7.50 ± 6.97	586.50^*^	0.042^*^
Amplitude in millivolts				
Mean ± SD	7.79 ± 4.99	6.84 ± 4.0	739.50	0.511
Nerve conduction velocity in m/s				
Mean ± SD	41.14 ± 7.77	38.95 ± 6.87	689.50	0.268
Sensorimotor peripheral neuropathy	40 (61.5%)	18 (72%)	1.455	0.535
Severe peripheral neuropathy	23 (35.4%)	12 (48%)	1.224	0.542

On comparing the two groups regarding NLR, the CAN group had a higher mean ± SD of 2.0 ± 0.81 than the non-CAN group, which had a mean ± SD of 1.59 ± 0.64, and this was statistically significant (p = 0.014). Also, neutrophils were higher in the CAN group with a mean ± SD of 4.73 ± 1.82 (109/L) than in the non-CAN group (3.76 ± 1.46; 109/L), and this was statistically significant (p = 0.10) (Table 4). Also, PLR had a higher mean ± SD of 136.4 ± 50.71 in the CAN group than in the non-CAN group (125.1 ± 58.25) but this was not statistically significant (p = 0.180) (Table 4).

**Table 4:** Comparison between the CAN and non-CAN groups regarding CBC indices (n = 90). p: p-value for comparing the two studied groups; *: statistically significant at p ≤ 0.05. CAN: cardiac autonomic neuropathy; WBC: white blood cells; N/L: neutrophil-to-lymphocyte; P/L: platelet-to-lymphocyte.

	24-hour Holter ECG		U	P
	Non-CAN (n = 65)	CAN (n = 25)		
WBCs (thousands/cmm)				
Min-max	3.60-43.0	4.70-10.82	668.00	0.193
Mean ± SD	7.58 ± 4.87	7.60 ± 1.87		
Median (IQR)	6.80 (5.70-8.18)	7.70 (5.60-9.41)		
N/L ratio				
Min-max	0.75-4.18	0.35-3.60	539.00^*^	0.014^*^
Mean ± SD	1.59 ± 0.64	2.0 ± 0.81		
Median (IQR)	1.53 (1.10-1.85)	2.28 (1.44-2.50)		
P/L ratio				
Min-max	36.36-368.1	48.33-291.7	663.50	0.180
Mean ± SD	125.1 ± 58.25	136.4 ± 50.71		
Median (IQR)	110.4 (84.81-153.1)	138.9 (96.66-162.0)		
Neutrophil (10^9^/L)				
Min-max	1.24-8.43	1.22-9.00	2.638^*^	2.638^*^
Mean ± SD	3.76 ± 1.46	4.73 ± 1.82		
Median (IQR)	3.70 (2.75-4.70)	4.80 (3.90-6.10)		
Lymphocyte (10^9^/L)				
Min-max	0.69-5.50	1.20-3.57	0.033	0.033
Mean ± SD	2.49 ± 0.89	2.49 ± 0.65		
Median (IQR)	2.50 (1.88-2.90)	2.66 (2.0-3.0)		

The diagnostic value of NLR as a marker to detect CAN

**Figure 1:**
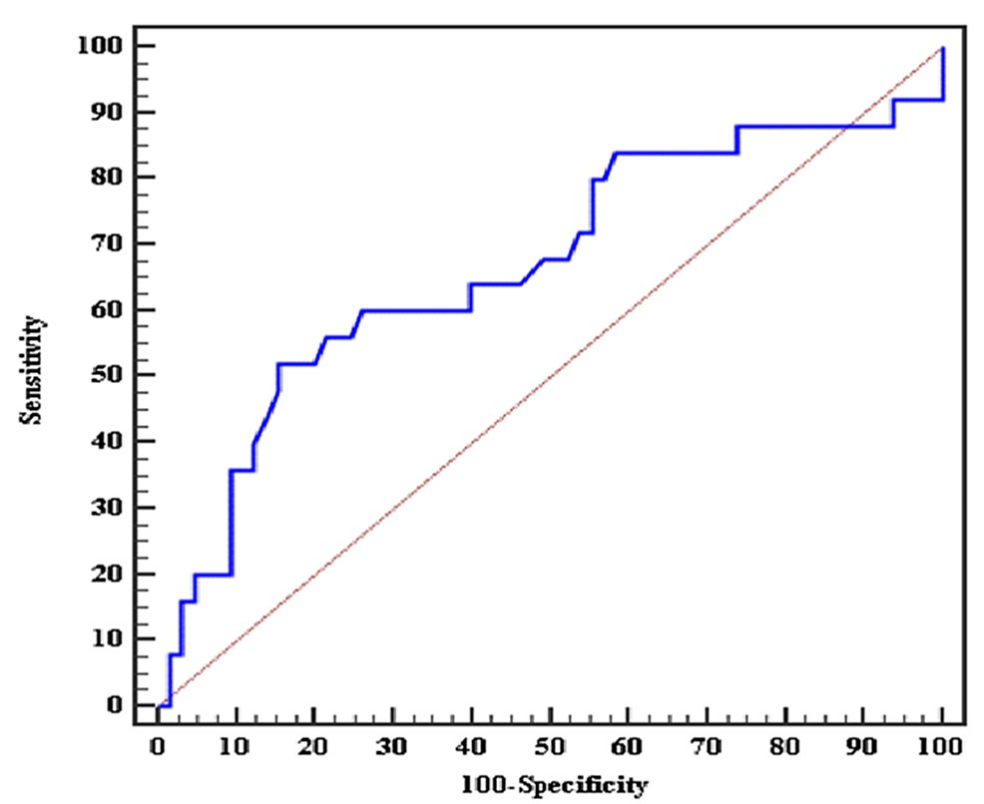
ROC curve for N/L ratio as a screening marker to detect CAN patients (n = 25) from non-CAN patients (n = 65). CAN: cardiac autonomic neuropathy; N/L: neutrophil-to-lymphocyte; ROC: receiver operating characteristics.

In the ROC analysis performed to find the optimal values of NLR for CAN development, a value of 1.7 was found optimal for NLR with 64% sensitivity and 60% specificity, and resulted in an area under the curve of 0.668 (Figure 1 and Table 5).

**Table 5:** N/L ratio as a screening marker to detect CAN patients (n = 25) from non-CAN patients (n = 65). *: Statistically significant at p ≤ 0.05. CAN: cardiac autonomic neuropathy; N/L: neutrophil-to-lymphocyte; AUC: area under the curve; p-value: probability value; NPV: negative predictive value; PPV: positive predictive value.

	AUC	p	95% CI	Cut-off	Sensitivity	Specificity	PPV	NPV
N/L ratio	0.668^*^	0.014^*^	0.532-0.805	>1.7	64.0	60.0	38.1	81.2

Relation between NLR and DPN

According to quantitative electrophysiological severity score for evaluation of the degree of neuropathy, our study showed that patients with severe DPN showed higher NLR mean ± SD of 1.80 ± 0.62 in comparison to mean ± SD of 1.62 ± 0.70 in the group of patients who had mild DPN but this was not statistically significant (Table 6).

**Table 6:** Relationship between DPN severity and CBC indices (n = 90). p: p-value for comparing between different categories. CAN: cardiac autonomic neuropathy; DPN: diabetic peripheral neuropathy; WBC: white blood cells; N/L: neutrophil-to-lymphocyte; P/L: platelet-to-lymphocyte.

	Severity			H	P
	Mild (n = 22)	Moderate (n = 33)	Severe (n = 35)		
WBCs (thousands/cmm)					
Mean ± SD	7.34 ± 1.88	8.48 ± 6.37	6.89 ± 2.34	4.043	0.132
Median (min-max)	7.2 (4.3-11.4)	7.6 (3.6-43.0)	6.1 (4.0-14.0)		
N/L ratio					
Mean ± SD	1.62 ± 0.70	1.66 ± 0.80	1.80 ± 0.62	2.196	0.334
Median (min-max)	1.5 (0.5-3.2)	1.6 (0.4-4.2)	1.8 (0.9-3.1)		
P/L ratio					
Mean ± SD	131.80 ± 52.17	127.14 ± 58.23	126.95 ± 58.19	0.266	0.875
Median (min-max)	123.2 (65.3-270.4)	108.1 (47.7-291.7)	125.6 (36.4-368.1)		
Neutrophil (10^9^/L)					
Mean ± SD	3.79 ± 1.20	3.96 ± 1.57	4.25 ± 1.88	0.590	0.557
Median (min-max)	3.9 (1.6-6.2)	3.7 (1.2-8.0)	3.9 (1.2-9.0)		
Lymphocyte (10^9^/L)					
Mean ± SD	2.52 ± 0.71	2.53 ± 0.71	2.43 ± 0.99	0.137	0.137
Median (min-max)	2.6 (1.4-4.0)	2.6 (1.2-4.1)	2.3 (0.7-5.5)		

## Discussion

Our study included 90 subjects, of whom 25 (27.8%) had CAN, diagnosed by decreased HRV by 24-hour Holter ECG. A study done in 1992 by Ziegler et al. discovered that 49% of patients with type 2 diabetes mellitus (T2DM) and DPN had CAN [18]. In our study, CAN patients with T2DM were associated with higher HbA1C (p = 0.005), similarly Pop-Busui showed that CAN was associated with poor glycemic control [19]. According to Zuern et al., patients with severe autonomic neuropathy had considerably higher HbA1c levels [20].

There was a significant positive correlation between NLR and CAN (p = 0.014), proving the potential involvement of immune imbalance and inflammation in the development of CAN.

The pathogenesis of CAN is due to multiple factors with different mechanisms that cause neuronal ischemia or direct neuronal death. The production of reactive oxygen species (ROS) and advanced glycation end products (AGEs) is increased by hyperglycemia [21].

The linking of the AGEs with their receptors (RAGE) forms a chronic cascade of inflammation and tissue injury, destroying the mechanisms for autonomic function and promoting the release of interleukin 6 and tumor necrosis factor α, which are pro-inflammatory cytokines, and vascular cell adhesion molecule 1, which increases the migration of leukocytes and neutrophil adhesion through the endothelium. Higher neutrophil levels are associated with the process of thrombus shape and ischemic injury [22].

In chronic inflammation, lymphocytes are expected to increase. On the contrary, lower lymphocyte levels in patients with T2DM occur as sustained hyperglycemia lowers interleukin-2 (IL-2) receptor expression levels due to CD25 deficiency. It is stated in a previous study that lymphocyte proliferation insufficiency (LPI) occurs in patients with diabetes [23].

The NLR is based on two markers: neutrophils (the initial line of defense) and lymphocytes (the regulatory component of inflammation). Several studies investigated NLR with blood glucose level regulation (HbA1C), and the results showed that NLR increases when HbA1C increases [24].

In our study, the ROC showed the cut-off point of NLR for the development of CAN is 1.7, while another study done in Indonesia on 57 subjects with T2DM showed a cut-off point of 1.34 [6]. This difference might be due to environmental, geographical, and lifestyle differences and also the need for a larger sample size study.

In the present study, results showed a higher level of PLR in the CAN group than in the non-CAN group. PLR has recently been used to have a predictive effect on diabetes mellitus and its complications. A cross-sectional study from Japan found that PLR can be a marker for high-risk diabetic foot and diabetic foot ulcers in patients with T2DM [25].

Our study showed higher NLR in the group who had severe DPN than in mild or moderate DPN, but this was not statistically significant. This finding is in line with Xu et al., who reported a positive correlation between NLR and DPN [9]. Another recent study by Chen et al. reported that NLR could be used as a prognosticator for DPN in T2DM [26]. On the contrary, a study done in India by Chittawar et al. showed different results, where the NLR was not a reliable prognosticator for the occurrence of DPN [27].

Many studies were done to show the relationship between NLR and diabetes complications, such as Öztürk et al., who conducted a study on 242 elderly patients with T2DM and discovered that patients with microvascular complications had higher NLR than patients without complications [28]. Another study by Singh et al. reported similar results, showing that the NLR could be considered a reliable predictive marker in diabetic nephropathy [29].

Several limitations should be considered when interpreting the findings of this study. The study design was cross-sectional, which may not be suitable for discovering the cause and effect between CAN and NLR; therefore, prospective longitudinal studies are essential to assess the prognostic significance of these ratios and their ability to predict the progression of CAN. Also, the study's single-center design and small sample size limit the generalization of our findings.

## Conclusions

There is a strong correlation between CAN and NLR, creating a possibility to use NLR value as a novel screening marker for CAN in patients with T2D. We succeeded in calculating the NLR cut-off point of 1.7 to detect CAN.
